# Bariatric Surgery Restores Cardiac and Sudomotor Autonomic C-Fiber Dysfunction towards Normal in Obese Subjects with Type 2 Diabetes

**DOI:** 10.1371/journal.pone.0154211

**Published:** 2016-05-03

**Authors:** Carolina M. Casellini, Henri K. Parson, Kim Hodges, Joshua F. Edwards, David C. Lieb, Stephen D. Wohlgemuth, Aaron I. Vinik

**Affiliations:** 1 Strelitz Diabetes Center for Endocrine and Metabolic Disorders and the Neuroendocrine Unit, Department of Medicine; Eastern Virginia Medical School, Norfolk, Virginia, United States of America; 2 Sentara Comprehensive Weight Loss Solutions, Sentara Medical Group, Norfolk, Virginia, United States of America; University of Hull, UNITED KINGDOM

## Abstract

**Objective:**

The aim was to evaluate the impact of bariatric surgery on cardiac and sudomotor autonomic C-fiber function in obese subjects with and without Type 2 diabetes mellitus (T2DM), using sudorimetry and heart rate variability (HRV) analysis.

**Method:**

Patients were evaluated at baseline, 4, 12 and 24 weeks after vertical sleeve gastrectomy or Roux-en-Y gastric bypass. All subjects were assessed using Sudoscan^TM^ to measure electrochemical skin conductance (ESC) of hands and feet, time and frequency domain analysis of HRV, Neurologic Impairment Scores of lower legs (NIS-LL), quantitative sensory tests (QST) and sural nerve conduction studies.

**Results:**

Seventy subjects completed up to 24-weeks of follow-up (24 non-T2DM, 29 pre-DM and 17 T2DM). ESC of feet improved significantly towards normal in T2DM subjects (Baseline = 56.71±3.98 vs 12-weeks = 62.69±3.71 vs 24-weeks = 70.13±2.88, p<0.005). HRV improved significantly in T2DM subjects (Baseline sdNN (sample difference of the beat to beat (NN) variability) = 32.53±4.28 vs 12-weeks = 44.94±4.18 vs 24-weeks = 49.71±5.19, p<0,001 and baseline rmsSD (root mean square of the difference of successive R-R intervals) = 23.88±4.67 vs 12-weeks = 38.06±5.39 vs 24-weeks = 43.0±6.25, p<0.0005). Basal heart rate (HR) improved significantly in all groups, as did weight, body mass index (BMI), percent body fat, waist circumference and high-density lipoprotein (HDL). Glycated hemoglobin (HbA1C), insulin and HOMA2-IR (homeostatic model assessment) levels improved significantly in pre-DM and T2DM subjects. On multiple linear regression analysis, feet ESC improvement was independently associated with A1C, insulin and HOMA2-IR levels at baseline, and improvement in A1C at 24 weeks, after adjusting for age, gender and ethnicity. Sudomotor function improvement was not associated with baseline weight, BMI, % body fat or lipid levels. Improvement in basal HR was also independently associated with A1C, insulin and HOMA2-IR levels at baseline.

**Conclusion:**

This study shows that bariatric surgery can restore both cardiac and sudomotor autonomic C-fiber dysfunction in subjects with diabetes, potentially impacting morbidity and mortality.

## Introduction

Obesity has become a global epidemic and rates continue to increase, generating a secondary increase in the risk for type 2 diabetes mellitus (T2DM) and cardiovascular disease worldwide [1]. Bariatric surgery has shown to be highly effective in inducing sustained weight loss and diabetes remission, and in reducing cardiovascular events and mortality [2–12]. Roux en Y gastric bypass (RYGB) and vertical sleeve gastrectomy (VSG), among others, result in significant rates of diabetes remission that can persist for over 4 years after surgery [5–8, 13–15]. However, the exact mechanisms by which these interventions induce long-term remission are still under debate and are not directly associated to weight loss per se. Intensive research that has emerged in the last decade shows that the process might be multifactorial, involving endocrine and sensory functions of the gastrointestinal tract, satiety hormones and regulation of appetite in the brain, β-cell function, insulin sensitivity and energy expenditure [16, 17].

Obesity in humans has been associated with autonomic dysfunction and increased sympathetic activity [18–21]. Furthermore, some studies have shown that weight loss improves measures of heart rate variability (HRV) and autonomic imbalance after both dietary restriction [22–26] and surgical interventions [27–31].

Autonomic function is affected early in patients with diabetes and changes in cardiac and peripheral autonomic function have been shown to occur before the advent of traditional risk factors and markers of inflammation [32]. Furthermore, increased heart rate (HR) and cardiac autonomic dysfunction have recently emerged as major risk factors for the development of cardiovascular disease and diabetes [33, 34].

Cardiac autonomic function can be quantified by time and frequency dependent measures of HRV [35]. Sweat glands have a postganglionic sympathetic C-fiber innervation that is regulated by acetylcholine and neuro-peptidergic activity. The functional impairment of this system can be quantified by sudorimetry using the Sudoscan device that measures electrochemical skin conductance (ESC) of hands and feet [36–40]. Sudoscan has shown to be useful in the detection of peripheral and autonomic diabetic neuropathy as well as diabetic nephropathy [41–46].

We propose that the mechanisms by which bariatric surgery improves diabetes outcomes and cardiovascular mortality could be related, in part, to improvements in autonomic function. The aim of this study was to evaluate the impact of bariatric surgery on cardiac and sudomotor autonomic C-fiber function in obese subjects with and without type 2 diabetes, using measures of sudorimetry and HRV. We hypothesized that by changing the functionality of the gastrointestinal tract and drastically reducing obesity and adipose tissue, autonomic imbalance and c-fiber function would improve in obese subjects with dysglycemia.

## Methods

The study included a total of 100 obese subjects with different stages of glycemic control. Subjects were evaluated at baseline and at 4, 12 and 24 weeks after bariatric surgery. Here we present an interim analysis on the first 70 patients who have completed up to 24-weeks of follow-up. Laparoscopic RYGP or VSG was performed in all participants and the decision regarding the type of surgery was made clinically by the surgeons. Exclusion criteria included: type 1 DM, chronic autoimmune conditions requiring treatment with immunosuppressive agents or systemic corticosteroids, history of epilepsy, active hepatitis B or C, or HIV infection, history of chronic arrhythmia, myocardial infarction (within 6 months of study enrollment), implanted pacemaker, active tobacco use, chronic use of weight loss medications, sympathomimetics, or other medications that could interfere with HRV interpretation. The study was approved by the Eastern Virginia Medical School Institutional Review Board, and all patients signed informed consent before participation.

### Study Outcomes

The primary outcomes were change from baseline in sudomotor (feet ESC) and cardiac autonomic function measures (time and frequency dependent measures of HRV) 24 weeks after bariatric surgery. Secondary outcomes included changes in measures of somatic nerve function and changes in measures of glycemic control (HbA1C (glycated hemoglobin), insulin and HOMA-IR (homeostatic model assessment) levels)

The following measures were obtained at baseline, 4, 12, and 24 weeks after surgery:

### Anthropometric measures

Height, weight, body mass index (BMI, calculated as weight in kilograms divided by height in meters squared), waist (inches), hip (inches), waist/hip ratio, and percent body fat (Bioelectrical impedance analysis (BIA) with the Omron HBF306C handheld device)

### Quantitative Autonomic Function Tests and Heart Rate Variability (HRV)

After resting for 15 minutes, heart rate was continuously recorded for 15 minutes in a temperature-controlled room (22–24°C). Time and Frequency domain analysis of HRV was determined at rest for 5’ with the patient sitting and breathing at a controlled rate (15 breaths per minute), during deep breathing maneuvers and during Valsalva maneuvers to measure sympathetic and parasympathetic function, and autonomic balance. These procedures have been previously described in detail [35]. Specifically sdNN (sample difference of the beat to beat (NN) variability), which is a measure of both sympathetic and parasympathetic action on HRV, and rmsSD (root-mean square of the difference of successive R-R intervals), a measure primarily of parasympathetic activity were obtained. Analysis of HRV was assessed using ANSAR (ANX 3.0 software; ANSAR Group, Inc., Philadelphia, PA).

### Sudomotor function

Sudoscan^TM^ (Impeto Medical, Paris, France) measures the capacity of the sweat glands to release chloride ions in response to electrochemical activation. A low-voltage (< 4V) galvanic current stimulates the underlying sweat glands, which generates a measurable flow of ions through the sweat ducts. Electrochemical Skin Conductance (ESC) of hands and feet are measured using 2 well-known principles: reverse iontophoresis and electrochemistry. ESC, expressed in micro-Siemens (μS), is the ratio between the current generated and the constant direct voltage stimulus applied between the electrodes. Measurement of ESC is dependent on the glands capability to transfer chloride ions and reflects small-C fiber function [38, 39]. During the test, patients were required to place their hands and feet on the electrodes and to stand still for 2–3 minutes. The device produces ESC results for individual right and left hands and feet. It then calculates an average score between right and left hands and feet. All the ESC results presented in this study correspond to the average ESC between right and left sides for both hands and feet. The reproducibility of these measurements has been validated in previous studies, and inter-device reproducibility has been confirmed through measurements with two different devices [40, 47].

### Neurological evaluation of the lower extremities

Neuropathy Impairment Score of the lower legs (NIS-LL), subdivided into sensory, reflex and motor components was determined in all patients; as previously described [48–50].

### Quantitative sensory testing (QST)

We used previously published methods and algorithms for measuring small fiber somatosensory function, including pressure perception, cold and warm thermal sensation detection thresholds, and heat-induced pain detection thresholds at the great toes [50]. These were assessed with the Q-Sense device (Medoc Advanced Medical, Minneapolis, MN). For each of these stimuli we applied the method of limits, 4 trials with an inter-stimulus interval randomly varying from 4 to 20 seconds. Thresholds were calculated as the mean stimulus intensity level over all 4 responses. Results are reported as mean threshold levels and delta (δ) from baseline, which is the difference between baseline and detection threshold temperature.

### Nerve conduction studies

Sural nerve amplitude potentials (SNAP) and conduction velocities were determined in the non-dominant leg using the DPN-Check devise (Neurometrix Inc., Waltham, MA) as previously described [51].

Blood samples were collected from all participants for determination of HbA1c, lipid profile including total serum cholesterol, HDL, LDL, triglycerides, and fatty acids, fasting plasma glucose, insulin, and C-peptide levels. Homeostatic model assessment-insulin resistance (HOMA-IR), Homeostatic model assessment-insulin sensitivity (HOMA%S) and Homeostatic model assessment β-cell function (HOMA%B) were calculated using the Oxford University on-line calculator (http://www.dtu.ox.ac.uk/homacalculator)[52, 53].

### Statistical Analysis

Continuous variables are expressed as means ± SEM (standard error of mean). Normal distribution of each continuous and categorical variable was confirmed by a normality test to ensure all appropriate assumptions were met for each statistical test used during analysis. Parametric (analysis of variance ANOVA) and non-parametric tests (Wilcoxon signed-rank test) were used to compare baseline mean differences between the groups, depending on sample size and distribution. If significance was observed, post hoc analysis was performed (Tukey-Kramer, Wilcoxon). Fisher's Exact Test was used for categorical variable. Repeated measures multivariate ANOVA (MANOVA) was used to analyze change from Baseline to 4, 12, and 24 weeks post-surgery on main outcome measures of autonomic function (HRV and ESC of feet and hands) and other secondary outcomes. ANOVA with post hoc analysis was used to determine between group differences at endpoint in primary outcome measures. Multivariate linear regression models were used to determine factors associated with improvement in primary outcome measures of autonomic function. All statistical analyses were performed using JMP Pro 10 statistical software (SAS Institute Inc., NC), with the risk of Type I error set at α = 0.05.

## Results

We included 70 obese subjects with different stages of glycemic control (24 non-DM, 29 pre-DM and 17 T2DM as defined by 2013 American Diabetes Association (ADA) guidelines)[54]. Baseline demographic characteristics of the 3 groups are shown in Table 1. As expected, subjects with T2DM had worse sudomotor and cardiac autonomic function together with worse neuropathy impairment scores and other measures of somatic nerve function, glucose, A1C, insulin, C-peptide levels and HOMA indexes. Subjects with pre-DM had significantly higher A1C levels when compared to non-DM subjects. VSG was performed in 56 subjects (80%) and RYGB in 14 subjects (20%).

**Table 1:** Baseline demographic characteristics.

	Non-DM* (n = 24)	Pre-DM*(n = 29)	T2DM* (n = 17)		p value**	
				T2DM vs Non-DM	T2DM vs Pre-DM	Pre-DM vs Non-DM
**Age**	41.23±2.36	39.71±2.20	52.252±2.41	**<0.01**	**<0.005**	0.99
**Gender F/M (%)**	19/5 (79/21)	25/4 (86/14)	13/4 (77/23)	0.73	0.66	0.62
**Ethnicity C/AA (%)**	17/7(71/29)	16/13(55/45)	8/9 (47/53)	0.29	0.54	0.27
**Surgery Type SG/RYGB**	20/4(83/17)	23/6 (79/21)	13/4 (76/24)	0.85	0.91	0.86
**Weight (Kg)**	133.02±5.11	135.43±4.49	120.67±4.74	0.23	0.11	0.93
**Body Mass Index**	46.75±1.39	46.99±1.34	44.18±1.52	0.15	0.17	0.99
**% body fat**	46.93±0.82	46.39±0.81	45.72±1.16	0.46	0.85	0.55
**Waist (cm)**	128.32±3.20	127.41±3.18	129.21±2.97	0.93	0.54	0.87
**Waist/Hip ratio**	0.90±0.02	0.94±0.03	0.98±0.02	**<0.05**	0.07	0.62
**Systolic Blood Pressure**	125.58±2.71	131.46±2.18	127.24±2.60	0.69	0.26	0.10
**Diastolic Blood Pressure**	75.04±1.84	80.36±1.43	75.24±1.73	0.87	0.05	0.05
**Basal Heart Rate**	77.92±2.71	75.39±1.92	77.35±3.05	0.87	0.83	0.63
**Neuropathy Impairment Score of Lower legs (NIS-LL)**						
**NIS-LL Reflex + Sensory Score**	1.75±0.63	2.45±0.52	5.59±1.16	**<0.005**	**<0.05**	0.14
**NIS-LL Total Score**	1.75±0.63	2.52±0.54	5.71±1.14	**<0.005**	**<0.05**	0.13
**Sudomotor Function (Electrochemical skin conduction (ESC) of hands and feet)**						
**Feet ESC**	68.00±2.24	69.24±1.86	56.71±4.41	**<0.05**	**<0.05**	0.82
**Hands ESC**	61.84±2.87	59.45±2.62	53.18±4.24	0.09	0.22	0.51
**Quantitative Autonomic Function Testing (Time domain analysis of HRV)**						
**Baseline sdNN**	50.50±6.01	43.39±4.38	32.53±4.28	**<0.05**	0.07	0.39
**Baseline rmsSD**	34.59±5.55	31.21±3.96	23.88±4.67	0.11	0.13	0.70
**Deep breathing sdNN**	54.70±5.44	58.04±6.26	42.88±6.24	0.08	0.13	0.73
**Deep breathing rmsSD**	33.61±4.09	39.75±4.98	26.41±3.39	0.12	0.24	0.48
**Quantitative Sensory Testing (QST)**						
**Pressure DT (monofilament)**	2.64±0.16	2.72±0.14	3.35±0.31	**<0.05**	0.05	0.45
**Cool DT great toe**	26.72±0.58	25.30±0.55	24.19±0.92	**<0.05**	**<0.05**	0.25
**Warm DT great toe**	42.57±0.71	44.60±0.70	45.36±1.04	**<0.05**	0.10	0.29
**Nerve Conduction Study (DPN-Check)**						
**Sural Amplitude (mV)**	5.56±1.13	7.35±1.35	7.34±2.19	0.92	0.72	0.33
**Sural CV (m/sec)**	52.11±1.22	48.71±0.82	47.44±2.56	0.07	0.06	0.34
**Biochemistries**						
**Fasting Plasma Glucose (mg/dl)**	84.25±2.59	90.24±2.10	126.00±14.01	**<0.001**	**<0.01**	0.10
**Cholesterol Total (mg/dl)**	169.83±5.34	186.47±6.86	177.19±7.79	0.48	0.41	0.09
**Triglycerides (mg/dl)**	99.42±7.39	123.93±10.65	131.56±16.33	0.10	0.79	0.10
**HDL (mg/dl)**	47.96±2.59	47.36±2.39	50.19±2.34	0.47	0.16	0.82
**LDL (mg/dl)**	101.00±4.83	114.68±6.35	100.56±6.51	0.90	0.23	0.16
**Fatty Acid (mEq/L)**	0.86±0.09	0.85±0.10	0.92±0.17	0.91	0.88	0.70
**Hemoglobin A1C (%)**	5.37±0.04	5.91±0.04	7.09±0.39	**<0.0001**	**<0.0005**	**<0.0001**
**Insulin (uIU/mL)**	11.73±1.44	17.95±2.46	23.10±4.88	**<0.05**	0.50	0.14
**C Peptide (ng/mL)**	2.61±0.23	3.41±0.33	3.69±0.41	**<0.05**	0.70	0.10
**HOMA 2%B**	168.99±26.55	169.98±12.63	136.07±24.34	0.71	0.24	0.77
**HOMA 2%S**	82.48±14.77	60.64±7.12	48.34±8.59	**<0.05**	0.30	0.09
**HOMA 2 IR**	1.45±0.18	2.26±0.31	3.11±0.62	**<0.05**	0.30	0.09

*2013 American Diabetes Association guidelines Diabetes Care 2013

**ANOVA, Wilcoxon Rank Sums Test or Fisher's Exact Test.

C/AA = Caucasian / African-American

CV = conduction velocity

DM = diabetes mellitus

DT = detection threshold

F/M = female / male

RYGB = Roux-en-Y gastric bypass

SG = sleeve gastrectomy

T2DM = type 2 diabetes

DT = detection threshold

ESC = electrochemical skin conductance

HRV = heart rate variability

rmsSD = root mean square of the difference of successive R-R intervals

sdNN = sample difference of the beat to beat (NN) variability

**Fig 1:**
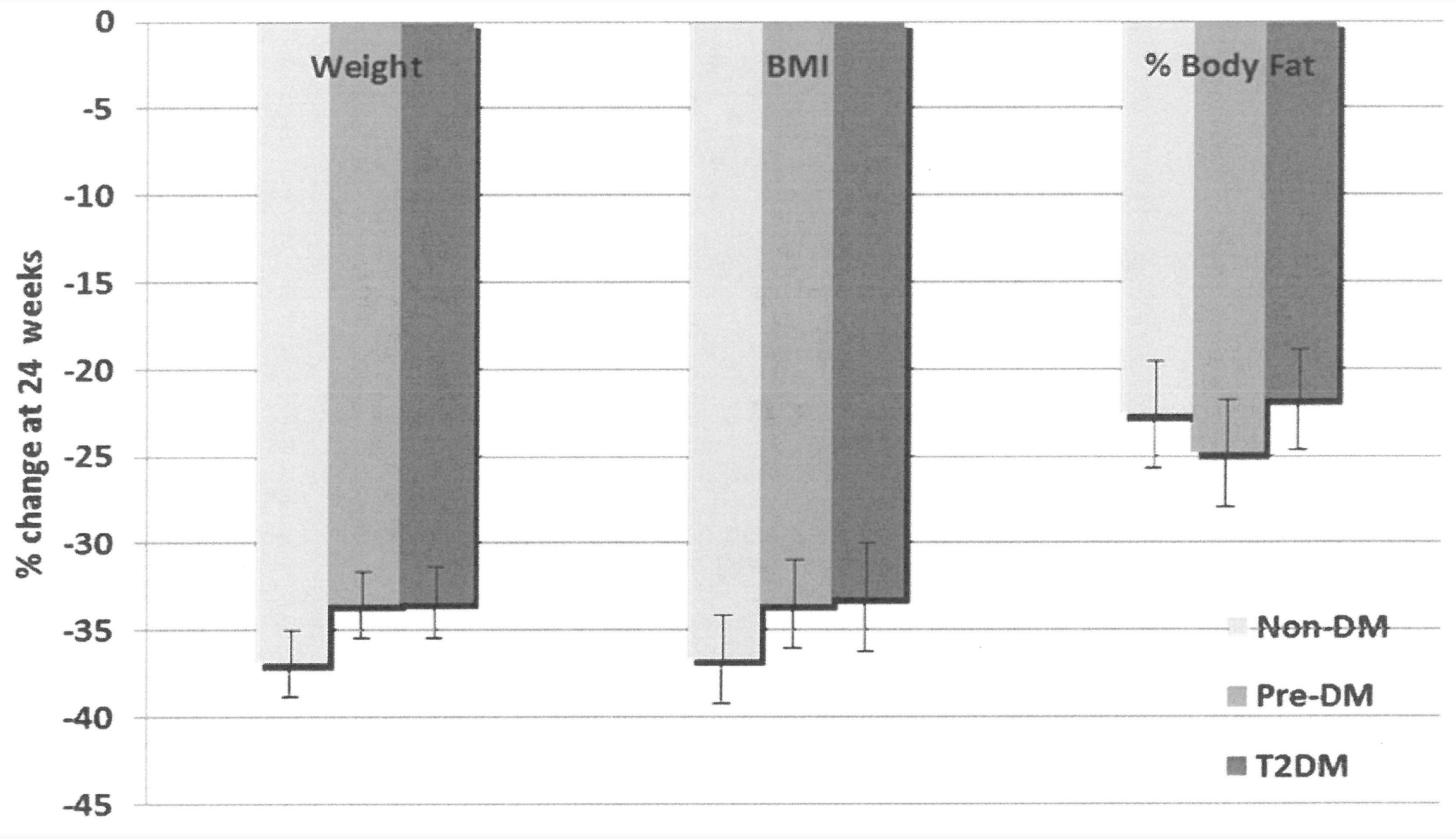
Changes in Anthropometric measures in the 3 groups after 24 weeks. Data presented as mean ± SEM. p = NS (ANOVA between group comparison at 24 weeks). DM = diabetes mellitus

**Fig 2:**
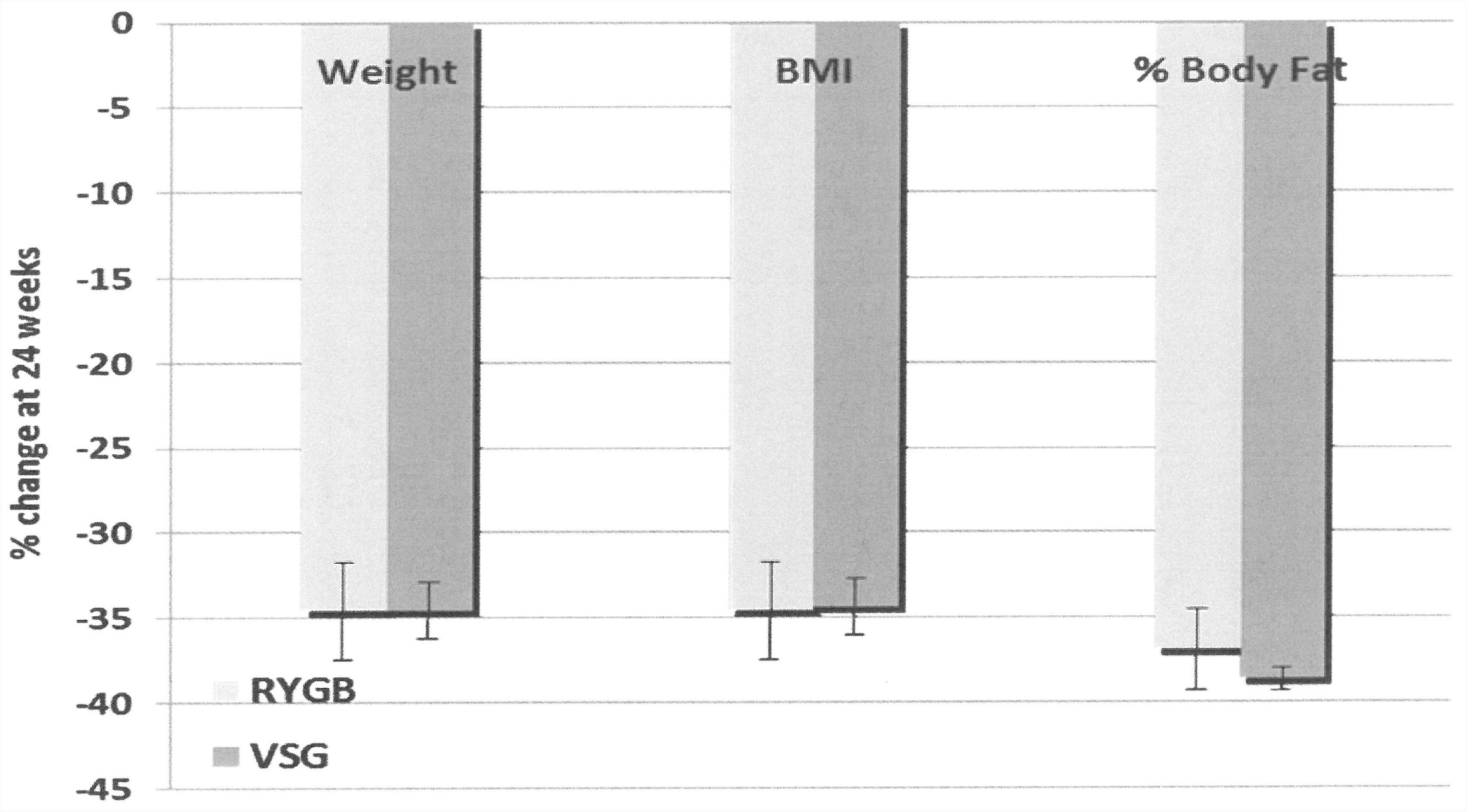
Changes in Anthropometric measures 24 weeks post roux-en-y gastric bypass or vertical sleeve gastrectomy. Data presented as mean ± SEM. p = NS (ANOVA between group comparison at 24 weeks). RYGB = roux-en-Y gastric bypass; VSG = vertical sleeve gastrectomy

Weight loss achieved at 24 weeks was similar in the 3 groups (% change from baseline: -36.90 in non-DM, -33.49 in pre-DM & -33.38 in T2DM subjects) as was % body fat (% change from baseline: -22.63 in non-DM, -24.82 in pre-DM & -21.72 in T2DM subjects) and BMI (Fig 1). These improvements were similar for both VSG and RYGB procedures (Fig 2).

**Fig 3:**
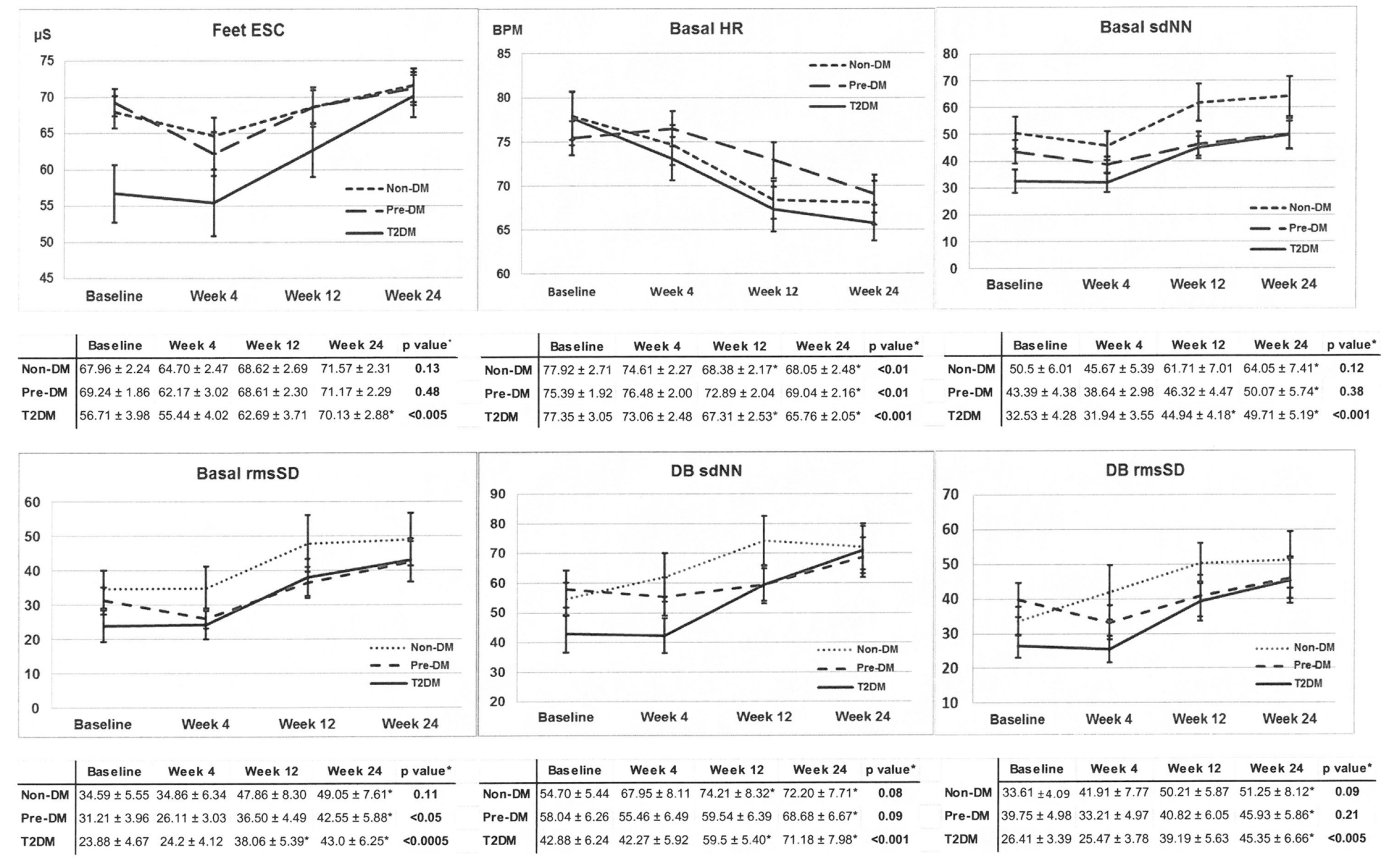
Change in primary endpoints 4, 12 and 24 weeks after bariatric surgery. *Repeated measures MANOVA. DB = deep breathing maneuver; DM = diabetes mellitus; ESC = electrochemical skin conductance; HR = heart rate; rmsSD = root mean square of the difference of successive R-R intervals; sdNN = sample difference of the beat to beat (NN) variability

Fig 3 shows sudomotor and cardiac autonomic function responses after 4, 12 and 24 weeks of surgery. Feet ESC improved significantly after 12 weeks and continued to improve at 24 weeks in the T2DM group but not in the other two groups *(within group comparison)*. HRV measures also improved significantly in T2DM only, with the exception of basal HR that improved in all 3 groups *(within group comparison)*. Type of surgical procedure (RYGB vs VSG) was included as a variable in the multivariate analysis done for each group. This was not found to be an independent factor influencing the improvement in sudomotor function (Table 2).

**Table 2:** Comparison of sudomotor response between different surgical procedures (RYGB vs VSG).

	Baseline Feet Mean ESC	W-12 Feet Mean ESC	W-24 Feet Mean ESC	P value*
**Non-DM**				
**RYGB**	71.34±1.38	76±1.54	78.34±1.45	0.611
**VSG**	66.19±1.68	66.69±1.95	69.44±1.77	
**Pre-DM**				
**RYGB**	68.17±2.21	62.17±2.36	70±2.14	0.258
**VSG**	69.77±1.87	70.36±2.06	71.41±2.18	
**T2DM**				
**RYGB**	46.75±3.22	62.5±2.91	64.75±2.53	0.197
**VSG**	61.36±2.06	65.55±2.15	70.73±2.16	

*Multivariate ANOVA (between group analysis RYGB vs VSG) DM = diabetes mellitus

ESC = electrochemical skin conductance

RYGB = Roux en Y Gastric Bypass

VSG = Vertical Sleeve Gastrectomy

The anthropometric, metabolic and somatic nerve function results before and after surgery, are shown in Tables 3 & 4. Anthropometric measures improved significantly in all 3 groups, with the exception of waist/hip ratio that was only significantly reduced in the T2DM group. Systolic blood pressure was reduced significantly in non-DM & pre-DM subjects. Measures of somatic nerve dysfunction did not show significant improvements in any of the groups. Measures of glycemic control and insulin resistance significantly improved after bariatric surgery in subjects with diabetes and pre-diabetes. HDL increased significantly in all groups and fatty acid levels decreased significantly in pre-DM & non-DM groups.

**Table 3:** Metabolic Measures after 4, 12 and 24 weeks of bariatric surgery.

	Baseline	4 weeks	12 weeks	24 weeks	p value*
**Non-Diabetes**					
**Weight (Kg)**	133.02±5.11	116.41±6.13	105.81±4.68	97.21±4.03	**<0.0001**
**Body Mass Index**	46.75±1.39	42.30±1.37	37.38±1.30	34.50±1.07	**<0.0001**
**% body fat**	46.93±0.82	43.84±1.02	41.54±1.22	38.95±0.95	**<0.0001**
**Waist (cm)**	128.35±3.20	119.18±3.23	108.10±5.23	103.86±3.07	**0.0001**
**Waist/Hip ratio**	0.90±0.02	0.88±0.02	0.85±0.03	0.86±0.01	0.37
**Systolic Blood Pressure**	125.58±2.71	121.13±2.56	117.00±2.46	118.86±2.75	**<0.005**
**Diastolic Blood Pressure**	75.04±1.84	75.83±1.87	73.10±1.83	74.27±2.16	0.70
**Fasting Plasma Glucose (mg/dl)**	84.25±2.59	89.58±5.12	86.81±1.93	84.40±1.81	0.65
**Cholesterol Total (mg/dl)**	169.83±5.34	160.62±11.57	176.44±11.35	169.62±8.03	0.49
**Triglycerides (mg/dl)**	99.42±7.39	88.85±7.43	100.31±7.54	94.57±8.24	0.06
**HDL (mg/dl)**	47.96±2.59	42.85±2.98	46.19±2.05	51.68±2.31	**<0.01**
**LDL (mg/dl)**	101.00±4.83	100.08±11.17	110.19±9.65	99.16±7.39	0.45
**Fatty Acid (mEq/L)**	0.86±0.09	0.81±0.08	0.55±0.06	0.47±0.07	**<0.05**
**Hemoglobin A1C (%)**	5.37±0.04	5.19±0.07	5.26±0.07	5.31±0.07	0.78
**Insulin (uIU/mL)**	11.73±1.44	10.26±1.22	11.11±2.64	8.11±0.81	**<0.05**
**C Peptide (ng/mL)**	2.61±0.23	2.74±0.19	3.05±0.41	2.69±0.20	0.71
**HOMA 2%B**	169.00±26.55	140.56±	125.37±15.70	109.92±7.21	**<0.001**
**HOMA 2%S**	81.78±13.77	76.66±8.37	99.71±12.42	115.03±12.45	**<0.05**
**HOMA 2 IR**	1.54±0.17	1.49±0.18	1.45±0.34	1.02±0.11	**<0.05**
**Pre-Diabetes**					
**Weight (Kg)**	135.42±4.49	121.88±4.35	113.47±4.313	102.68±4.18	**<0.0001**
**Body Mass Index**	46.98±1.34	42.32±1.37	39.67±1.35	35.67±1.36	**<0.0001**
**% body fat**	46.39±0.81	43.23±1.03	40.87±1.17	37.87±1.29	**<0.0001**
**Waist (cm)**	127.41±3.18	121.44±3.12	115.57±2.80	108.48±3.05	**<0.0001**
**Waist/Hip ratio**	0.94±0.03	0.91±0.02	0.90±0.02	0.90±0.02	0.16
**Systolic Blood Pressure**	131.46±2.18	126.33±2.18	126.00±2.88	129.66±2.18	**<0.05**
**Diastolic Blood Pressure**	80.36±1.43	79.00±1.92	79.25±1.48	80.76±1.52	0.85
**Fasting Plasma Glucose (mg/dl)**	90.24±2.10	89.06±2.39	88.12±1.85	88.19±1.33	0.72
**Cholesterol Total (mg/dl)**	186.46±6.86	168.28±6.51	181.35±6.80	190.24±6.59	0.81
**Triglycerides (mg/dl)**	123.93±10.65	109.39±9.78	110.59±9.86	94.89±6.52	0.05
**HDL (mg/dl)**	47.36 ±.39	38.72±1.53	48.53±2.27	55.88±2.33	**<0.01**
**LDL (mg/dl)**	114.68±6.35	107.61±6.48	110.82±5.81	115.44±6.44	0.71
**Fatty Acid (mEq/L)**	0.85±0.10	0.81±0.06	0.58±0.07	0.51±0.07	0.08
**Hemoglobin A1C (%)**	5.91±0.04	5.68±0.08	5.41±0.07	5.53±0.05	**<0.001**
**Insulin (uIU/mL)**	17.95±2.46	15.36±2.36	11.98±1.42	9.35±1.45	**<0.05**
**C Peptide (ng/mL)**	3.41±0.33	3.28±0.21	2.99±0.28	2.58±0.23	**<0.05**
**HOMA 2%B**	169.98±12.63	161.22±11.59	132.87±10.44	108.56±10.14	**<0.001**
**HOMA 2%S**	60.64±7.12	60.13±7.72	77.31±7.57	116.08±14.05	**<0.01**
**HOMA 2 IR**	2.26±0.31	2.24±0.35	1.53±0.18	1.20±0.18	**<0.01**
**Type 2 Diabetes**					
**Weight (Kg)**	120.67±4.75	106.59±4.65	98.42±4.48	91.48±4.56	**<0.0001**
**Body Mass Index**	44.18±1.52	39.21±1.56	36.19±1.50	33.56±1.55	**<0.0001**
**% body fat**	45.72±1.16	42.02±1.36	40.15±1.45	38.11±1.67	**<0.0001**
**Waist (cm)**	129.21±2.97	121.49±3.48	111.84±3.18	106.22±3.45	**<0.0001**
**Waist/Hip ratio**	0.98 0.02	0.95 0.02	0.92 0.02	0.92 0.02	**0.0001**
**Systolic Blood Pressure**	127.24±2.60	126.63±3.36	126.50±3.91	125.59±3.44	0.81
**Diastolic Blood Pressure**	75.24±1.73	78.38±2.63	75.75±2.66	76.47±2.27	0.90
**Fasting Plasma Glucose (mg/dl)**	126.00±14.01	123.43±12.76	119.00±11.08	96.20±4.39	**<0.01**
**Cholesterol Total (mg/dl)**	177.19±7.79	175.71±8.87	181.29±14.33	174.67±13.30	0.70
**Triglycerides (mg/dl)**	131.56±16.33	128.00±23.95	125.29±14.38	107.13±12.60	0.05
**HDL (mg/dl)**	50.19±2.34	45.00±4.28	51.29±3.32	58.07±3.03	**<0.01**
**LDL (mg/dl)**	100.56±6.51	104.86±6.57	104.71±12.47	106.27±5.32	0.84
**Fatty Acid (mEq/L)**	0.92±0.17	0.66±0.11	0.70±0.12	0.70±0.10	**<0.05**
**Hemoglobin A1C (%)**	7.09±0.40	6.71±0.44	6.55±0.29	6.15±0.19	**<0.001**
**Insulin (uIU/mL)**	23.10±4.88	17.10±2.09	18.23±4.80	9.55±1.87	**<0.001**
**C Peptide (ng/mL)**	3.69±0.41	4.44±0.46	3.83±0.57	2.63±0.36	**<0.05**
**HOMA 2%B**	136.07±24.34	108.96±13.42	127.23±18.61	97.43±13.42	**<0.001**
**HOMA 2%S**	48.34±8.59	41.13±6.07	54.27±10.31	102.24±25.48	**<0.001**
**HOMA 2 IR**	3.11±0.62	2.76±0.39	2.39±0.62	1.35±0.27	**<0.001**

*Repeated measures MANOVA. HOMA = Homeostasis model assessment

**Table 4:** Somatic Neuropathy Measures after 4, 12 and 24 weeks of surgery in obese subjects with and without T2DM.

	Baseline	4 weeks	12 weeks	24 weeks	p value*
**Non-Diabetes**					
**NIS-LL Motor Score**	0	0	0	0	NA
**NIS-LL Reflex + Sensory Score**	1.75±063	1.70±0.73	1.34±0.28	0.86±012	0.33
**NIS-LL Total Score**	1.75±0.63	1.78±0.48	1.34±0.50	0.86±0.35	0.33
**Pressure DT (monofilament)**	2.64±0.16	2.64±0.14	2.50±0.17	2.47±0.17	0.30
**Cool DT great toe**	26.72±0.58	27.36±0.56	27.08±0.60	26.31±0.63	0.24
**Warm DT great toe**	42.57±0.71	42.20±0.86	43.46±0.87	42.82±0.80	0.59
**Sural Amplitude (mV)**	5.56±1.13	6.38±1.03	5.38±0.74	5.68±0.65	0.05
**Sural CV (m/sec)**	52.11±1.22	50.75±2.05	53.06±1.86	53.44±1.39	0.38
**Pre-Diabetes**					
**NIS-LL Motor Score**	0.07±0.05	0.24±0.15	0.07±0.05	0.07±0.05	0.92
**NIS-LL Reflex + Sensory Score**	2.45±0.52	2.69±0.60	2.29±0.55	2.41±0.62	0.74
**NIS-LL Total Score**	2.52±0.54	2.93±0.62	2.36±0.56	2.48±0.62	0.74
**Pressure DT (monofilament)**	2.72±0.14	2.66±0.17	2.62±0.18	2.55±0.17	0.18
**Cool DT great toe**	25.30±0.55	26.23±0.42	26.40±0.51	25.810.58	0.10
**Warm DT great toe**	44.60±0.70	44.48±0.71	44.61±0.71	43.70±0.83	0.39
**Sural Amplitude (mV)**	7.35±1.35	6.00±1.27	6.17±0.70	8.11±1.22	0.07
**Sural CV (m/sec)**	48.71±0.82	47.59±1.97	50.06±0.99	50.37±1.21	0.50
**Type 2 Diabetes**					
**NIS-LL Motor Score**	0.12±012	0	0	0	0.91
**NIS-LL Reflex + Sensory Score**	5.59±1.17	4.81±1.05	4.06±1.19	4.69±1.34	0.18
**NIS-LL Total Score**	5.71±1.14	4.81±1.05	4.06±1.19	4.69±1.34	0.18
**Pressure DT (monofilament)**	3.35±0.31	3.26±0.20	3.03±0.32	2.84±0.27	**<0.05**
**Cool DT great toe**	24.19±0.92	24.59±0.96	24.76±0.74	24.05±0.78	0.52
**Warm DT great toe**	45.36±1.04	44.01±1.32	44.61±1.16	45.64±0.92	0.64
**Sural Amplitude (mV)**	7.33±2.19	8.29±2.01	7.67±1.43	8.50±2.01	0.50
**Sural CV (m/sec)**	47.44±2.56	48.86±3.07	50.33±2.25	49.67±1.48	0.55

*Repeated measures MANOVA. CV = conduction velocity; DT = detection threshold; mV = microvolts; m/sec = meters per second; NIS-LL = neurologic impairment score of the lower legs.

**Fig 4:**
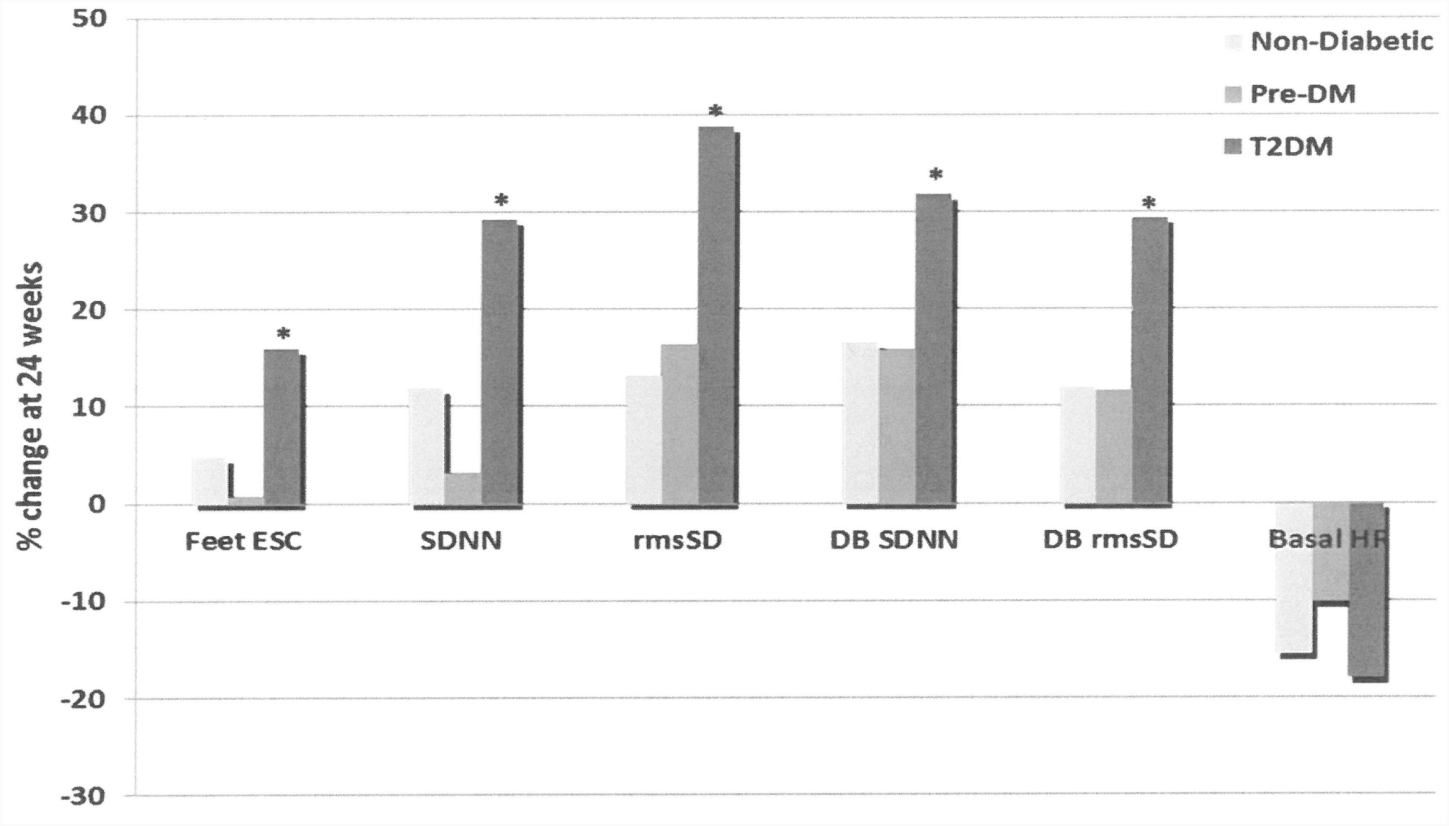
Changes in Sudomotor and Cardiac autonomic measures in the 3 groups. Data presented as mean ± SEM *p<0.05 (ANOVA between group comparison at 24 weeks). DB = deep breathing maneuvers; DM = diabetes mellitus; ESC = electrochemical skin conductance; HR = heart rate; HRV = heart rate variability; rmsSD = root mean square of the difference of successive R-R intervals; sdNN = sample difference of the beat to beat (NN) variability

When comparing improvements in primary endpoints, T2DM group % change in measures of sudomotor and cardiac autonomic function were significantly better that those observed in non- and pre-DM groups (between group comparison) (Fig 4).

**Fig 5:**
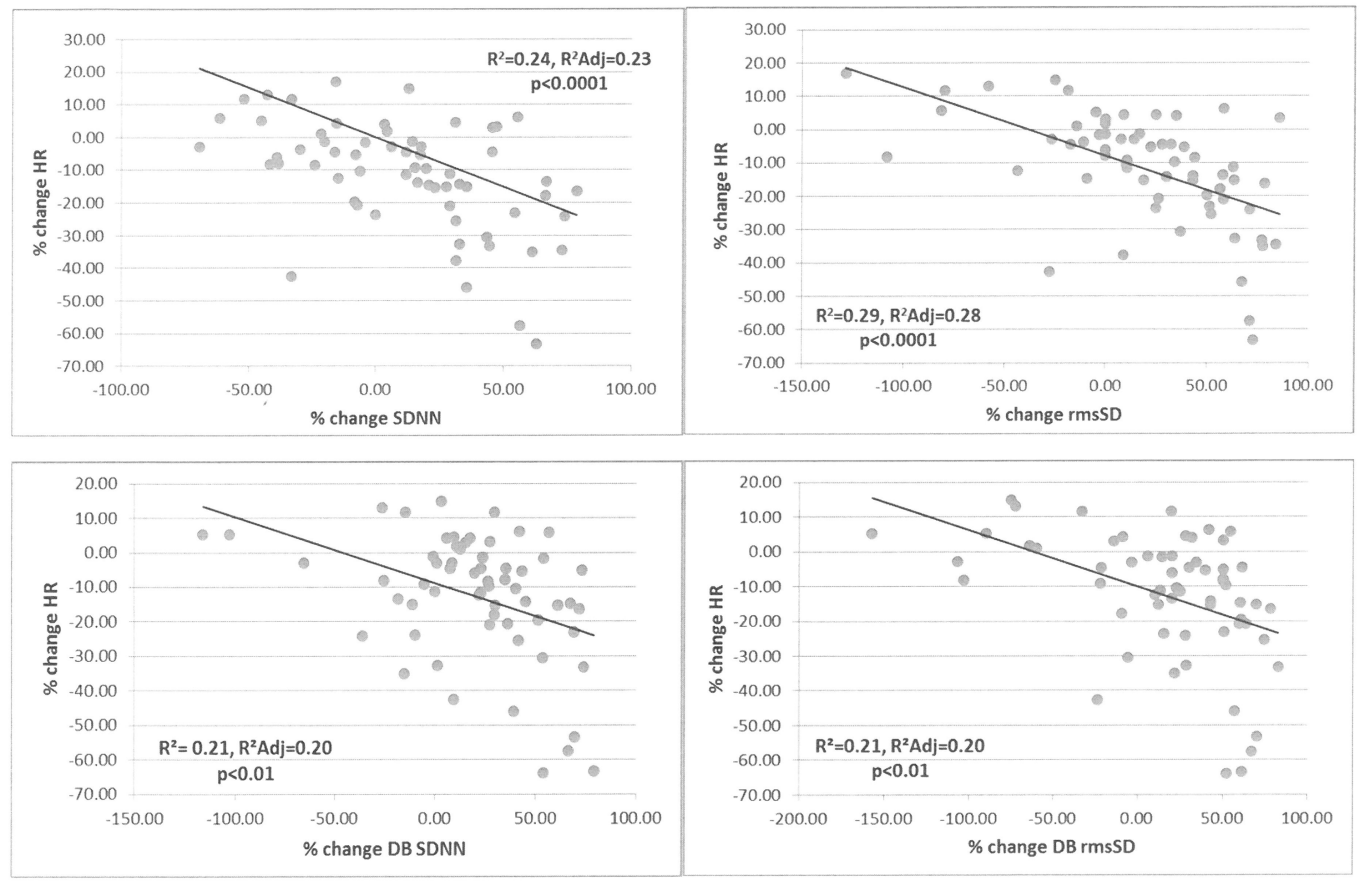
Correlations between improvements in heart rate with improvements in HRV measures 24 weeks after bariatric surgery. DB = deep breathing maneuvers; HR = heart rate; HRV = heart rate variability; rmsSD = root mean square of the difference of successive R-R intervals; sdNN = sample difference of the beat to beat (NN) variability

On multiple linear regression analysis, feet ESC improvement at 24 weeks was independently associated with HbA1C, insulin and HOMA2-IR levels at baseline, and improvement in HbA1C at 24 weeks; after adjusting for age, gender and ethnicity. Sudomotor function improvement was not associated with baseline weight, BMI, % body fat or lipid levels at 24 weeks. Improvements in basal HR were also independently associated with HbA1C, insulin and HOMA2-IR levels at baseline, after adjusting for age, gender and ethnicity (Table 5). Percent change in basal HR correlated significantly with percent change in all HRV measures, after adjusting for age, gender, ethnicity and presence of DM (Fig 5).

**Table 5:** Association between improvements in sudomotor autonomic function and heart rate, and measures of glycemic and metabolic control.

	Covariables	R^2^	R^2^ Adj	p value*
**Feet ESC (% change)**	**A1C**	0.2861	0.1991	0.007
	**Insulin**	0.2554	0.1765	0.009
	**HOMA-IR**	0.2931	0.1993	0.005
	**Weight**	0.1709	0.0653	0.932
	**BMI**	0.1349	0.0568	0.848
	**% body fat**	0.1779	0.0743	0.699
**Basal HR (% change)**	**A1C**	0.3317	0.2522	0.005
	**Insulin**	0.3487	0.2644	0.004
	**HOMA-IR**	0.3554	0.2685	0.004
	**Weight**	0.129	0.024	0.218
	**BMI**	0.126	0.021	0.217
	**% body fat**	0.115	0.015	0.826

* Multivariate linear regression models. Adjusted for age, gender and ethnicity.

A1C = glycated hemoglobin

BMI = body mass index

ESC = electrochemical skin conductance

HOMA-IR = homeostasis model assessment-Insulin Resistance

HR = heart rate

At study entry 14(88%) T2DM subjects were receiving metformin, 4 (25%) incretin-based therapies (GLP-1 analogs or DPP4 inhibitors), 3 (18%) sulfonylureas, 2(12%) thiazolidinediones and 2(12%) basal insulin. After 24 weeks only 2 patients (12%) were still on treatment with metformin. All other treatments were stopped. Adverse events were mild, transient and uncommon (<10%) and included food intolerance, nausea, headaches, cold intolerance, hair loss, fatigue, constipation and dizziness.

## Discussion

This analysis shows that bariatric surgery significantly improves sudomotor and cardiac autonomic function in obese subjects with diabetes after 24 weeks. As expected weight, BMI, percent body fat and lipid levels improved in subjects with and without diabetes. Measures of glycemic control and insulin resistance improved in subjects with diabetes and pre-diabetes. In this cohort, improvement of sudomotor and cardiac autonomic functions were independently associated with the presence of DM, HbA1C, insulin and HOMA2-IR levels at baseline, and with improvement in HbA1C levels; but was not associated with improvement in weight, BMI, % body fat or lipid levels.

Even though several studies have shown improvements in cardiac autonomic function, to our knowledge the relationship between bariatric surgery and sudomotor C-fiber function in subjects with diabetes has not been reported before. Our group and others have previously shown that feet ESC has a high sensitivity and specificity for the detection of diabetic neuropathy [41, 43, 45]. Reductions in ESC also correlate with the presence of diabetic kidney disease [42, 44, 46]. Furthermore, measures of HRV correlated significantly with feet ESC in different cohorts [41, 45]. The results of this report show, for the first time, the clinical utility of Sudoscan as an endpoint measure in interventional studies.

Obesity is associated with reduced vagal function, increased sympathetic activity and sympathovagal imbalance [18, 20, 21, 55]. Hormonal changes in obesity such as insulin resistance, hyperinsulinemia, and hyperleptinemia have been implicated in the development of autonomic dysfunction in the obese state [56, 57]. Diet-induced weight loss improves HRV measures and autonomic imbalance in both obese otherwise healthy individuals and obese T2DM subjects [22–26]. Recent studies have also reported on the effects of surgical interventions on measures of autonomic dysfunction [27–31, 58]. Improvements in measures of HRV were reported in all these studies, both in non-DM as well as T2DM participants [59–61]. However, these were either small, non-controlled studies or included few or no subjects with T2DM. Consensus regarding whether the improvements in autonomic function are related to improvements in other metabolic parameters is less clear. Maser and colleagues evaluated 32 obese patients (8 with T2DM) before and after RYGB. Similar to other groups, HRV measures increased significantly after 6 months, although this was independent from improvements in insulin resistance measures, weight and BMI [29]. Similar findings have been reported by others [30, 62]. Other studies have shown a direct association between changes in weight, BMI, waist circumference, insulin resistance measures, cholesterol and triglyceride levels, and changes in cardiac autonomic function [31, 61]. Our results show that improvements in both cardiac and sudomotor autonomic function are independently associated with measures of insulin resistance and glycemic control but not with weight, BMI or body fat.

This report demonstrates for the first time the effects of bariatric surgery on measures of sudomotor function in obese subjects with pre-DM. The lack of improvement in autonomic function measures in our group of subjects with pre-DM is surprising. We have previously shown that both autonomic and somatic nerve dysfunction occur early in dysglycemia, even before the diagnosis of DM [32, 63]. In this cohort, subjects with pre-DM had worse autonomic function measures when compared to non-DM participants. However, significant improvements in cardiac and sudomotor dysfunction were not seen in this group, despite the fact that insulin resistance measures did improve after surgery. It may be that the number of non-DM and pre-DM patients was insufficiently powered for the changes in cardiac autonomic function to reach significance. Nonetheless, subjects with pre-DM clearly differ from T2DM subjects and, accordingly, behave differently. These findings warrant further investigation.

It has been consistently shown that cardiac autonomic dysfunction is an independent risk factor for cardiovascular disease (CVD). In a post hoc analysis of two large cohorts of patients with stable chronic CVD (ONTARGET & TRANSCEND), resting baseline and in-trial average HR were independently associated with significant increases in cardiovascular events and all-cause mortality [34]. Wulsin and colleagues [33] examined the contribution of two measures of autonomic imbalance, resting heart rate and HRV, on the development of CVD, diabetes, and early mortality. Both measures, along with sex, age, and smoking were significant predictors for the development of CVD, DM and early mortality within 12 years in the Framingham Heart Study offspring cohort. This reinforces the importance of our findings and those of other groups on the effects of bariatric surgery in cardiac and sudomotor function. Furthermore, significant correlations were found between improvements in HR and other measures of HRV in our cohort (see Fig 5).

The significant changes in autonomic function that were not associated with weight, BMI, body fat, or lipid level changes suggest an independent mechanism and a probable link between the gastrointestinal tract, pancreatic β-cell function, insulin sensitivity and the hypothalamus, which involves GLP-1 and other hormones of the GI tract in the regulation of energy homeostasis via the central nervous system. Evaluation of inflammatory markers and intestinal hormones in conjunction with changes in autonomic function after bariatric surgery will help in further elucidating these mechanisms.

In contrast with the improvement in small fiber sudomotor function, measures of large fiber somatic nerve dysfunction did not show significant improvements after bariatric surgery. This is not surprising since the duration of the study (six months) is insufficient for recovery of myelinated nerve fibers, which in general take 2 or more years, presumably because of the slow rate of remyelination [64, 65].

The main limitation of our study is the lack of a control, non-surgical group. However, previous studies have consistently shown that bariatric surgery is significantly more effective than lifestyle interventions in restoring metabolic changes and improving microvascular outcomes in obese subjects with DM [2, 3, 5–8, 11–14]. We acknowledge that, at the time of this report, the number of patients in each group is not equally distributed, with a smaller number of participants in the T2DM group. Nonetheless it was the latter that achieved significant change. We are also aware that 20% of the patients underwent RYGB, which differs from VSG. Current data suggest that glucose metabolism improvements occur earlier (before significant weight loss) and are more prominent in patients receiving RYGB vs. VSG or banding [2, 66]. Consequently this could have had different effects on glucose metabolism and sudomotor function in our cohort. However, a recent report from the Swedish Obese Subjects (SOS) study showed that improvements in glucose metabolism were not independently associated with the type of surgery performed [67]. Table 2 shows that sudomotor function improvement did not differ significantly between the two surgical procedures for each of the 3 groups on this report. Nonetheless we acknowledge that the statistical results could have been affected in this subgroup analysis due to the small sample size. In conclusion, this report shows that bariatric surgery can restore both cardiac and sudomotor autonomic C-fiber dysfunction towards normal in subjects with diabetes, potentially impacting morbidity and mortality. Results of in depth studies on GI hormones, inflammatory markers and cytokines will further elaborate on these findings, and improve our understanding of the underling mechanisms by which bariatric surgery improves cardiovascular disease and diabetes.
